# Combining microTESE and trifocal TESE improves sperm retrieval and cryopreservation outcomes in nonobstructive azoospermia

**DOI:** 10.1007/s11255-026-05037-z

**Published:** 2026-02-10

**Authors:** Shimi Barda, Noga Fuchs Weizman, Ron Hauser, Haggar Zoe Aspitz, Hadar Amir, Yael Kalma, Ofer Lehavi, Sandra Edith Kleiman, Foad Azem, Snir Dekalo

**Affiliations:** 1 Institute for the Study of Fertility, Lis Maternity Hospital, Tel Aviv Sourasky Medical Center, 6 Weizmann Street, 6423906 Tel Aviv, Israel; 2 Racine IVF Unit, Lis Maternity Hospital, Tel Aviv Sourasky Medical Center, Tel Aviv, Israel; 3 Department of Urology, Tel Aviv Sourasky Medical Center, Tel Aviv, Israel; 4 Gray Faculty of Medical & Health Sciences, Tel Aviv University, Tel Aviv, Israel; 5 Israel Academic College, Ramat Gan, Israel; 6 The New York State/American Program, Faculty of Medicine, Tel Aviv University, Tel Aviv, Israel

**Keywords:** Azoospermia, Sperm retrieval, Cryopreservation, Assisted reproductive techniques, Male infertility

## Abstract

**Purpose:**

To evaluate whether combining microdissection testicular sperm extraction (microTESE) with trifocal testicular sperm extraction (TESE) in a single session improves sperm retrieval and cryopreservation outcomes in men with nonobstructive azoospermia.

**Materials and methods:**

Retrospective cohort study of 93 men with nonobstructive azoospermia (out of 176 consecutive men with azoospermia) who underwent both microTESE and trifocal TESE sequentially during the same procedure was analyzed. Specimens from each technique were processed and analyzed separately to enable within-patient comparison. Sperm retrieval rates, quantitative sperm yield, and cryopreserved vials were measured. Histopathological patterns were classified. Statistical significance was set at *p* < 0.05.

**Results:**

Sperm retrieval was successful in 59 of 93 patients, resulting in an overall retrieval rate of 63.4%. When analyzed separately, spermatozoa were identified in microTESE specimens from 54 patients (58.1%) and in trifocal TESE specimens from 51 patients (54.8%). The combined approach (analyzing all specimens from both techniques) achieved a significantly higher retrieval rate (63.4%) than either technique analyzed alone (*p* = 0.041 vs. microTESE; *p* = 0.002 vs. trifocal TESE). In seven patients (7.5%), sperm were found exclusively in microTESE specimens, and in three patients (3.2%), exclusively in trifocal specimens. Quantitative sperm yield was also higher with the combined approach (mean ± SD: 9.9 ± 5.1 sperm units) than with either microTESE (3.9 ± 3.2) or trifocal TESE (6.0 ± 3.4) specimens alone (*p* < 0.001). This resulted in more cryopreserved vials (12.4 ± 2.4 vs. 5.9 ± 1.2, *p* < 0.001), enabling greater fertility preservation potential. Histopathological analysis revealed a mixed atrophy pattern in all successful retrievals, supporting the biological rationale for combining targeted and systematic sampling in patients with focal spermatogenesis.

**Conclusion:**

A dual technique approach combining microTESE and trifocal TESE significantly improves sperm retrieval rates and cryopreservation outcomes in nonobstructive azoospermia compared to either technique alone. This strategy maximizes fertility preservation and may reduce the need for repeat testicular procedures.

## Introduction

Azoospermia, defined as the complete absence of spermatozoa in the ejaculate, affects approximately 1% of all men and up to 15% of infertile males worldwide [1, 2]. Nonobstructive azoospermia (NOA) represents the most challenging subgroup, as spermatogenesis is typically severely impaired and focal in distribution. For these patients, surgical sperm retrieval remains the only option for achieving biological parenthood. Over the past 3 decades, several surgical techniques have been developed, ranging from conventional testicular sperm extraction (TESE) to advanced microsurgical methods. Despite these technological advances, sperm retrieval rates (SRR) remain highly variable, reflecting the focal and unpredictable nature of spermatogenesis in NOA [3, 4].

Microdissection TESE (microTESE), introduced in 1999, allows the surgeon to identify and selectively sample seminiferous tubules with a larger diameter and more opaque appearance, which are more likely to contain active spermatogenesis [5]. In contrast, trifocal TESE relies on systematic tissue sampling from three anatomically distinct regions of the testis (upper, mid, and lower poles), thereby increasing the likelihood of detecting scattered foci of spermatogenesis [6]. Each technique offers unique advantages but also inherent limitations: microTESE is more targeted but may miss foci outside the visualized field, whereas trifocal TESE provides broader sampling but less precision [7, 8].

Alongside advancements in surgical sperm retrieval, cryopreservation of testicular tissue has become a critical component of NOA management. Cryopreservation not only enables long-term storage of retrieved sperm, but also provides flexibility in coordinating ICSI cycles and avoiding repeat surgeries. The clinical implications of using fresh sperm obtained intraoperatively versus frozen–thawed sperm have been widely investigated. While some studies suggest that fresh sperm may offer advantages in terms of motility and viability [9], recent evidence indicates comparable ICSI success rates between fresh and frozen–thawed spermatozoa [10–12]. These findings highlight the clinical importance of maximizing sperm retrieval and cryopreservation during a single procedure.

Given the importance of maximizing sperm retrieval in a single procedure, combining the precision of microTESE with the broader coverage of trifocal TESE may enhance the likelihood of retrieval success. While this concept was first suggested by Marconi et al. [6], additional support for this combined approach has been reported in subsequent studies, including Falcone et al. [13], which focused on low-chance retrieval NOA patients. The evidence base, however, remains limited. Existing studies focus primarily on sperm retrieval rate (SRR) and often lack quantitative analysis of sperm yield and cryopreservation outcomes. Furthermore, most published series are small, heterogeneous, or lack systematic within-subject comparisons.

The present study addresses these gaps by evaluating sperm retrieval outcomes in a large, single center cohort of NOA patients undergoing combined microTESE and trifocal TESE during the same surgical session. Specifically, we aim to quantify not only retrieval rates but also total sperm yield, cryopreservation outcomes, and subsequent ICSI results. This structured within-subject design allows for a direct comparison between techniques and provides new insight into their complementary value in clinical practice.

## Materials and methods

### Study design

The study was approved by the local Institutional Review Board (approval no. 01–262). We conducted a retrospective cohort analysis of a prospectively maintained database of consecutive men with azoospermia who were evaluated for surgical sperm retrieval between January 2018 and November 2024. This was a within-subject comparative study in which all participants underwent both microTESE and trifocal TESE sequentially during a single operative session. Each patient, therefore, served as his own control, allowing direct paired comparison of the two techniques. Specimens from each technique were processed separately, enabling assessment of their independent and combined contributions while controlling for inter-patient variability in testicular histology, hormonal profile, and genetic background. This approach provides key methodological advantages: elimination of inter-patient variability, increased statistical power through paired analysis, and direct evaluation of both techniques within the same testicular environment.

### Study participants

A total of 176 men with azoospermia were evaluated at our tertiary fertility center. All patients underwent a standardized preoperative evaluation that included detailed medical history, physical examination, hormonal profiling, karyotype analysis, and Y-chromosome microdeletion testing. Written informed consent was obtained from all participants. Following this evaluation, 58 patients (33.0%) were diagnosed with obstructive azoospermia and managed accordingly and were therefore excluded from this study. The remaining 118 men were diagnosed with nonobstructive azoospermia (NOA) according to the guidelines of the American Urological Association (AUA) and the American Society for Reproductive Medicine (ASRM). The diagnosis was established based on clinical and laboratory evaluation and supported by typical findings of serum follicle-stimulating hormone (FSH) levels ≥ 7.6 mIU/mL and reduced testicular volume (≤ 19 mL) [14]. Of these 118 NOA patients, 25 (21.2%) were excluded for the following reasons: AZFa or AZFb Y-chromosome microdeletions (*n* = 3, 2.5%); prior testicular surgery (*n* = 16, 13.6%); and severe testicular atrophy (bilateral testicular volume < 4 mL) with FSH > 40 mIU/mL, in which patients declined surgical intervention after counseling (*n* = 6, 5.1%).

The final study cohort comprised 93 men with confirmed NOA who underwent combined microTESE and trifocal TESE during the same surgical session, representing 78.8% of eligible NOA candidates.

### TESE surgical procedure

All surgeries were performed by senior microsurgeons with extensive experience in testicular sperm retrieval. A single midline scrotal incision was made to expose the testes. A transverse tunical incision allowed visualization of the parenchyma, and an operating microscope with × 20 magnification was used for the microTESE stage. This step was performed first to identify and selectively sample opaque and enlarged seminiferous tubules, which are more likely to harbor active spermatogenesis.

Immediately following completion of microTESE, a trifocal TESE was performed by excising tissue from three anatomically distinct regions: the upper pole, mid-portion, and lower pole of the testis. In the trifocal TESE component, each biopsy site yielded approximately 100–200 mg of testicular tissue, resulting in a total tissue amount of approximately 400–600 mg per testis. In the microTESE component, targeted sampling was performed under optical magnification, with each extracted fragment weighing approximately 5–10 mg, and an overall estimated tissue yield of 40–60 mg per testis, depending on intraoperative findings.

Samples from microTESE and each trifocal location were collected separately in pre-labeled containers for subsequent cytological processing. Hemostasis was achieved, the testis was closed in layers, and the procedure was repeated on the contralateral side.

### Tissue processing, sperm evaluation, and cryopreservation

Tissue samples were transferred immediately to the laboratory in modified human tubal fluid (HTF) medium and maintained at 37 °C. Each specimen was processed individually using a standardized protocol. Tissue was mechanically minced with two sterile 25-gauge needles for 3–5 min, vortexed for 10 s. After allowing larger tissue fragments to settle, the supernatant was collected for analysis. An initial 5 μL aliquot from the supernatant was examined by wet preparation under phase contrast microscopy (× 200) to determine the presence or absence of spermatozoa. To stimulate sperm motility prior to evaluation, GM501 SpermMobil (GYNEMED, Hamilton, Germany) was added to the sample according to the manufacturer’s instructions.

For quantitative assessment, twelve separate 5 μL drops were sequentially examined under an inverted microscope (× 200). A “Sperm unit” was defined as the total number of spermatozoa (motile and immotile) counted across all 12 droplets from a single specimen, representing an absolute count rather than a concentration. Each biopsy specimen obtained by microTESE and trifocal TESE was processed and analyzed separately to allow paired, within-subject comparison between techniques.

Quantification was standardized using an identical microscopic processing and counting protocol for all specimens, rather than by normalization to tissue weight, due to the inherent variability in fragment size between targeted microTESE sampling and systematic trifocal biopsies.

Whenever spermatozoa were identified, the remaining suspension was cryopreserved using standard cryoprotectants (Test-Yolk Buffer, Irvine Scientific, USA), with a maximum of 15 cryovials per patient. Sperm unit counts and number of cryopreserved vials were recorded separately for each biopsy site and surgical technique.

### Histopathology assessment

For histopathological analysis, a single tissue fragment was obtained from the mid-testicular region of each testis, separate from the tissue used for microTESE and trifocal TESE sperm retrieval, and specifically designated for pathological evaluation. These specimens were fixed in Bouin’s solution, embedded in paraffin, and stained with hematoxylin and eosin. A minimum of 20 seminiferous tubules were examined per specimen. Histopathological classification was determined by the most advanced germ cell stage identified and was categorized as normal spermatogenesis, mixed atrophy, early maturation arrest (spermatocyte stage), late maturation arrest (round spermatid stage), or Sertoli cell-only pattern. All slides were initially evaluated by the andrology laboratory and subsequently verified by the pathology department to ensure diagnostic consistency between observers. This specimen was not intended to represent all sampled regions of the testis, but was selected to allow standardized histological classification while preserving tissue for sperm retrieval.

### Statistical analysis

Descriptive statistics were used to characterize demographic, hormonal, cytological, and histological variables. Given the within-subject paired design, comparisons between microTESE and trifocal TESE outcomes were performed using the Friedman test for continuous variables and Cochran’s Q test for categorical variables, with post hoc Dunn’s test and Bonferroni correction for multiple comparisons when significant. Pearson’s Chi-square test was used for univariate analysis of categorical variables. Continuous variables were analyzed using one-way ANOVA or the independent *t *test for parametric distributions and the Wilcoxon rank-sum test for nonparametric distributions. Proportions and rates were reported with 95% confidence intervals (CI) calculated using the Wald method. All statistical tests were two-tailed, and a *p* value of < 0.05 was considered statistically significant.

## Results

A total of 93 men with nonobstructive azoospermia underwent combined trifocal TESE and microTESE procedures. Spermatozoa were successfully retrieved in 59 patients, yielding an overall sperm retrieval rate (SRR) of 63.4% (95% CI 53.7–73.2), while no spermatozoa were retrieved in 34 patients (36.6%).

Table 1 summarizes the demographic and clinical characteristics of the cohort stratified by retrieval outcome. There were no statistically significant differences in age, serum FSH levels, karyotype, Y-chromosome microdeletions, or testicular dimensions between men with successful and unsuccessful sperm retrieval (all *p* > 0.05). No baseline variable was significantly associated with retrieval outcome.

**Table 1:** Characteristics of the study cohort stratified by men with successful sperm retrieval vs. retrieval failure

Variable	Successful sperm retrieval (*n* = 59)	Retrieval failure (*n* = 34)	*p* value
Age (years)	32.4 ± 3.8	34.1 ± 5.8	0.28
FSH (mIU/mL)	25.9 ± 9.2	22.4 ± 4.9	0.12
Klinefelter syndrome 47, XXY	10 (17%)	5(15%)	0.79
AZFc microdeletion	0 (0%)	1 (3%)	0.37
Right testicular diameter (cm)	3.7 ± 0.8	3.8 ± 0.7	0.39
Left testicular diameter (cm)	3.6 ± 0.9	3.6 ± 1.1	0.42

Continuous variables are presented as mean ± SD; categorical variables as n (%). *p *values were calculated using independent *t *test or Chi-square/Fisher’s exact test as appropriate. AZFc = azoospermia factor c (Y-chromosome microdeletion)

Histopathological analysis demonstrated a mixed atrophy pattern in all successful retrieval cases (*n* = 59, 100%). Among the 34 patients with unsuccessful retrieval, Sertoli cell-only pattern was observed in 25 cases (73.5%) and maturation arrest was observed in 9 cases (26.5%).

### Sperm retrieval outcomes by technique

To evaluate the relative effectiveness of the different retrieval strategies, SRR was calculated separately for microTESE, trifocal TESE, and the combined approach. The combined technique achieved an SRR of 63.4% (95% CI 53.7–73.2), which was significantly higher than the SRR observed with trifocal TESE alone (54.8%, 95% CI 44.7–65.0; *p *= 0.002) and microTESE alone (58.1%, 95% CI 48.0–68.1; *p* = 0.041). Within-patient analysis demonstrated that in seven men (11.9% of successful cases; 7.5% of the entire cohort), spermatozoa were retrieved exclusively from microTESE specimens, whereas in three patients (5.1% of successful cases; 3.2% of the entire cohort) spermatozoa were identified only in trifocal TESE samples.

### Quantitative sperm yield and cryopreservation

Among patients with successful retrieval (*n* = 59), the mean (± SD) sperm yield from microTESE was 3.9 ± 3.2 sperm units, compared with 6.0 ± 3.4 sperm units from trifocal TESE and 9.9 ± 5.1 sperm units using the combined approach (*p* < 0.001, Friedman test).

There was a strong relationship between sperm yield and cryopreservation outcomes. In cases where spermatozoa were retrieved from both techniques (*n* = 49), an average of 12.4 ± 2.4 vials were cryopreserved, and 27 patients (46%) reached the maximum of 15 vials. When spermatozoa were retrieved from only one technique (*n* = 10), significantly fewer vials were stored (5.9 ± 1.2 vials; *p* < 0.001).

Overall, combining microTESE and trifocal TESE significantly increased both sperm retrieval rates and total sperm yield compared with either technique alone, highlighting their complementary value in NOA management.

## Discussion

Nonobstructive azoospermia (NOA), characterized by minimal to absent spermatogenesis, remains a significant clinical challenge. Its etiology includes genetic disorders (e.g., sex chromosomal abnormalities, translocations, and Y-chromosome microdeletions), anatomic conditions (e.g., cryptorchidism, post-testicular torsion state), and iatrogenic factors (e.g., radiation exposure and gonadotoxic agents) [15]. Given the heterogeneity of NOA, SRR vary widely across patient populations and surgical extraction methods [3, 4, 16, 17]. In this study, we systematically compared sperm retrieval outcomes within the same NOA patient using microTESE, trifocal TESE, and a combined approach—a design that minimizes interindividual variability and strengthens the validity of the comparison. Our findings demonstrate that combining microTESE with trifocal TESE not only improves SRR, but also yields a significantly higher number of retrieved spermatozoa. This increased yield directly translated to a greater number of cryopreserved vials per patient, potentially enabling multiple ICSI cycles and reducing the need for repeat surgical interventions. These results highlight the clinical advantage of an integrated approach in optimizing fertility treatment strategies for men with NOA.

In our cohort of men with NOA, the combined microTESE and trifocal TESE technique proved to be significantly more efficient than either technique alone, with SRR of 63% compared to 58% for microTESE alone and 55% for trifocal TESE alone. Several previous studies have assessed the efficacy of microTESE and conventional TESE separately, reporting variable retrieval rates depending on patient characteristics, histopathological findings, and surgical expertise [7, 8]. However, direct within-subject comparisons remain scarce. Marconi et al. [6] were among the first to systematically evaluate different TESE techniques in NOA patients, demonstrating a potential benefit to combining approaches, although their cohort was small and quantitative sperm yield was not assessed.

Our findings are broadly consistent with prior reports supporting the complementary value of combining targeted microsurgical sampling with broader regional sampling, as also suggested in the low-chance retrieval series by Falcone et al. [13]. Unlike our within-subject design, Falcone et al. compared outcomes across different patient groups, but their results similarly support the additive benefit of the combined approach.

Indirect evidence from other studies further supports the notion that microTESE enhances retrieval outcomes, particularly in patients with Sertoli cell-only or maturation arrest patterns [4]. Our data extends this concept by demonstrating that combining microTESE with trifocal TESE not only increases SRR, but also enhances sperm yield, particularly in cases where sperm was retrieved exclusively by one technique but not the other (11.9% by microTESE alone and 5.1% by trifocal TESE alone). This within-subject comparison underscores the complementary nature of these techniques and provides a more robust basis for clinical decision making.

MicroTESE provides the distinct advantage of direct visualization of testicular tissue, allowing surgeons to target enlarged, opaque tubules that are more likely to contain spermatogenesis. This targeted approach explains why microTESE succeeded in cases where trifocal TESE failed. Conversely, conventional trifocal TESE employs systematic sampling of different testicular regions, which occasionally detected spermatozoa in areas missed by the more focused microTESE approach. Despite the higher precision of microTESE, it remains debatable whether this technique alone offers sufficient benefit to justify its increased complexity and cost [3]. Our results suggest that a hybrid approach maximizes sperm retrieval by capitalizing on the complementary strengths of both techniques, the microsurgical precision of microTESE and the broader sampling approach of trifocal TESE.

### Factors affecting sperm retrieval

Given the heterogeneity of NOA, various studies have explored predictive factors for sperm retrieval success, including clinical, hormonal, and genetic parameters. Some studies suggest that FSH levels, testicular volume, and prior response to hormonal treatments may influence retrieval outcomes [18–21]. However, the only well-established genetic predictor remains Y-chromosome microdeletions in the AZFa and AZFb regions, where sperm retrieval is virtually impossible [22]. In our cohort, no clinical or laboratory variable was identified as an independent predictor of successful sperm retrieval (Table 1), further reinforcing the importance of the chosen retrieval technique in determining outcomes.

### Cryopreservation and future ICSI cycles

Our study further highlights the practical advantages of increased sperm retrieval, as greater sperm yield directly correlated with the number of cryopreserved vials. The mean number of spermatozoa retrieved using the combined approach (9.9 ± 5.1 sperm units) was significantly higher than with either microTESE (3.9 ± 3.2) or trifocal TESE (6.0 ± 3.4) alone (*p* < 0.001). This translated to an average of 12.4 ± 2.4 cryopreserved vials when sperm was retrieved from both techniques, compared to only 5.9 ± 1.2 vials when retrieved from a single technique. The availability of multiple vials provides a greater reservoir of sperm for future use, potentially increasing the number of ICSI cycles available per patient without requiring additional surgeries, a significant benefit considering the invasive nature and potential complications of testicular procedures.

### Limitations

Several limitations of our study should be acknowledged. First, we did not have precise data on the weight of each biopsy sample or on post-surgical testosterone levels.

MicroTESE alone is known to involve smaller tissue samples, theoretically reducing the risk of postoperative hypogonadism. However, previous studies have demonstrated that testosterone levels generally return to baseline following both microTESE and conventional TESE [23–25], and we found no clinical evidence of long-term hypogonadism in our cohort. In addition, sperm yield was not normalized to exact tissue weight, as fragment size varied substantially between targeted microTESE sampling (approximately 10–20 mg per fragment) and systematic trifocal biopsies (approximately 100–200 mg per site); therefore, quantification relied on standardized microscopic processing and a paired within-subject design rather than tissue-based normalization. Second, while our study included a consecutive cohort of NOA patients, its retrospective nature may introduce selection bias. Third, our quantification method using “sperm units” based on sperm count in multiple drops, while standardized within our study, may differ from methods used in other centers, potentially limiting direct comparisons of quantitative outcomes across studies. Fourth, histopathology was performed only on mid-testicular specimens to preserve tissue for sperm retrieval and cryopreservation while still obtaining standardized histology. Although this limits regional mapping, the heterogeneity seen within mid-testicular samples supports the rationale for combining targeted and systematic sampling, as focal spermatogenesis may be scattered throughout the testis.

## Conclusion

Our results support the combined microTESE and trifocal TESE approach as a superior strategy for sperm retrieval in NOA patients. This technique not only improves SRR, but also increases the quantity of retrieved spermatozoa and the number of cryopreserved vials, potentially allowing for multiple ICSI cycles without requiring repeat surgical interventions. Future research should focus on long-term reproductive outcomes and explore the cost effectiveness of different retrieval strategies in NOA management.

## Data Availability

The data that support the findings of this study are available from the corresponding author upon reasonable request.
